# Investigation of Seasonal Variation in Fatty Acid and Mineral Concentrations of Pecorino Romano PDO Cheese: Imputation of Missing Values for Enhanced Classification and Metabolic Profile Reconstruction

**DOI:** 10.3390/metabo13070877

**Published:** 2023-07-24

**Authors:** Leonardo Sibono, Massimiliano Grosso, Stefania Tronci, Massimiliano Errico, Margherita Addis, Monica Vacca, Cristina Manis, Pierluigi Caboni

**Affiliations:** 1 Department of Mechanical, Chemical and Materials Engineering, University of Cagliari, Via Marengo 2, 09123 Cagliari, Italy; leonardo.sibono@unica.it (L.S.); stefania.tronci@unica.it (S.T.); 2 Department of Green Technology, University of Southern Denmark, Campusvej 55, 5230 Odense, Denmark; maer@igt.sdu.dk; 3 Agris Sardegna, Servizio Ricerca Prodotti di Origine Animale, Agris Sardegna, Loc., Bonassai, 07040 Sassari, Italy; maddis@agrisricerca.it; 4 Servizio Ricerca Studi Ambientali, Difesa delle Colture e Qualità delle Produzioni, Viale Trieste, 09123 Cagliari, Italy; mvacca@agrisricerca.it; 5 Dipartimento di Scienze della vita e Ambiente, Cittadella Universitaria di Monserrato Blocco A, 09012 Monserrato, Italy; cristina.manis@unica.it (C.M.); caboni@unica.it (P.C.)

**Keywords:** metabolomics, cheese seasonality, Pecorino Romano PDO, conjugated linoleic acids, Omega-3, fatty acids, mineral, probabilistic principal component analysis, missing data, linear discriminant analysis, cross validation

## Abstract

Seasonal variation in fatty acids and minerals concentrations was investigated through the analysis of Pecorino Romano cheese samples collected in January, April, and June. A fraction of samples contained missing values in their fatty acid profiles. Probabilistic principal component analysis, coupled with Linear Discriminant Analysis, was employed to classify cheese samples on a production season basis while accounting for missing data and quantifying the missing fatty acid concentrations for the samples in which they were absent. The levels of rumenic acid, vaccenic acid, and omega-3 compounds were positively correlated with the spring season, while the length of the saturated fatty acids increased throughout the production seasons. Concerning the classification performances, the optimal number of principal components (i.e., 5) achieved an accuracy in cross-validation equal to 98%. Then, when the model was tasked with imputing the lacking fatty acid concentration values, the optimal number of principal components resulted in an *R*^2^ value in cross-validation of 99.53%.

## 1. Introduction

In the context of increasingly complex industrial processes characterized by several highly correlated variables, common univariate analysis approaches often fail to describe a system’s characteristics comprehensively. The introduction of multivariate statistical analysis has received significant attention in several fields in the process industry, such as quality control, fault detection, process monitoring, process control, and predictive modelling [1,2].

Within the framework of the food industry, increasing consumer awareness of food quality and safety has led to a growing demand for the development of healthier products [3,4], thus driving the food sector toward the implementation of improved technological and manufacturing techniques [5]. Specifically, dairy manufacturers are focused on monitoring and controlling the levels of lipids, proteins, minerals, and vitamins in their products, as these compounds strongly impact human health [6].

Different studies have reported that sheep’s milk cheese could have a beneficial effect on human health due to the presence of bioactive compounds such as conjugated linoleic acids (CLA, e.g., *C18:2 cis 9*, *trans 11*), vaccenic acid (*C18:1 trans 11*), and omega-3 family compounds, which play important roles as nutraceutical substances [7,8]. The concentration of these nutritional compounds in cheese products is known to vary significantly throughout different cheese production seasons owing to many factors, such as changes in animal feed fatty acid composition and lactation stages [7]. Therefore, developing an effective classification tool for cheese samples to study the effect of a specific stimulus or treatment, such as seasonality, on the properties of the cheese is imperative for ensuring the control of product characteristics. To date, metabolomics has been a powerful discipline with respect to analyzing food products, especially dairy products, as it enables the employment of a variety of analytical techniques together with statistical tools, thus facilitating the assessment of product quality and safety for several food matrices [9,10,11]. Principal Component Analysis (PCA) is a commonly used multivariate technique for reducing data dimensionality [12] owing to its simplicity and effectiveness in identifying the variables that primarily contribute to explaining data variability in a high-dimensional data matrix. Nevertheless, due to its lack of a probabilistic formulation, PCA cannot handle datasets with missing data [13]. The challenge of managing missing data in the food industry remains an issue of concern across various food matrices, such as pasta [14] and tea [15].

Missing data are ubiquitous in food processes since only a subset of product properties of interest can be generally observed because of cost, time, or technical limitations [14,16]. Such missing information may significantly impair modelling performance. In these cases, when employing a traditional modelling approach, the only available method for handling missing data is discarding observations with incomplete information, which results in a loss of valuable data and hinders the prediction of characteristics that might otherwise be inferred in an analysis [17].

Probabilistic Principal Component Analysis (PPCA) is a multivariate model that addresses some of the limitations of its deterministic formulation, as it provides the ability to reconstruct a data matrix wherever missing data are present while retaining a model parameter solution equivalent to that obtained from the conventional formulation [18]. In addition, PPCA offers a more robust classification model thanks to its probabilistic approach, which enables a better assessment of the uncertainty in the resulting model response [19]. To the best of the authors’ knowledge, the study of the impact of seasonality on the nutritional properties of cheese through modelling methods capable of dealing with missing data appears to be lacking.

This study aims to expand the current advancements in food-omics by addressing the presence of missing values via investigating the influence of seasonality on the fatty acid and mineral profiles of Pecorino Romano cheese samples. PPCA coupled with Linear Discriminant Analysis (LDA) was first employed to classify cheese samples produced during winter (January), mid-spring (April), and early summer (June) and identify the variables responsible for such classifications. Then, the model was employed in reconstructing the missing information. The analysis reported in this study is presented from a comparative perspective, as the PPCA model’s performance was compared with more consolidated approaches, namely, Partial Least Squares Discriminant Analysis (PLS-DA) in the case of a classification problem and Partial Least Squares Regression (PLSR) in the case of the inference of unknown data values [20]. The robustness of the models was evaluated using a hybrid cross-external validation approach for classification and Montecarlo cross-validation for missing data inference.

## 2. Materials and Methods

The dataset examined in this study was obtained from Pecorino Romano PDO cheese samples produced in 12 dairies situated in Sardinia (Italy) over three distinct months, namely, January, April, and June. The ripening time was 8 months. Once the percentage of Fatty Acid Methyl Esters (% FAME) and mineral concentration were experimentally determined, a 45 × 81 matrix was obtained. Specifically, 73 variables were related to % FAME, and the remaining 8 corresponded to minerals (Ca, Mg, Na, K, P, S, Zn, and Fe). A total of 45 samples were analyzed, of which 33 offered complete % FAME and mineral concentration information, while the remaining 12 lacked this information. In more detail, regarding January and April, there were 24 samples (12 per production month) for which both the % FAME and mineral concentration were known. Hereafter, these samples will be referred to as “complete samples”. In addition to these complete samples, four samples per month lacked % FAME information, and these samples constituted the missing data series of the experimental data matrix. Hereafter, these samples will be referred to as “partial samples”, as only their mineral content is known. Similarly, there are nine complete samples for June along with six additional partial samples. Thus, dataset is balanced for all three seasons.

### 2.1. Analytical Methods

#### 2.1.1. Cheese Composition and Nitrogen Fractions

Cheese samples were analyzed with respect to pH (using pH meter 420 A, Orion, Boston, MA, USA), dry matter (DM) [21], fat (using the Soxhlet method [22]), total nitrogen (TN) [23], protein (TN*6.38), soluble nitrogen (SN) at pH 4.6, soluble nitrogen in 12% trichloroacetic acid (SN-TCA), soluble nitrogen in 10% phosphotungstic acid (SN-PTA) [24], NaCl [25] (ISO 5943-IDF 88, 2006), and ash. To determine the ash content, cheese samples (5 g) were pre-dried at 102 °C for 24 h and then calcined at 550 °C in a muffle furnace (Gelman Instrument, Opera, Italy). The operating conditions were set to reach 550 °C in 8 h and then maintain 550 °C for another 8 h [26]. In this work, the effect of the cheese production season on the macro-components was studied first.

#### 2.1.2. Fatty Acid Methyl Ester Analysis

The fatty acid profiles of the cheese samples were determined according to the analytical procedure reported in [7]. In brief, FAMEs were obtained through the basic trans-methylation of the cheese fat extracted according to the methodology reported by Jiang et al. [27] and analyzed using a gas chromatograph coupled with a flame ionization detector (GC-FID). Each fatty acid methyl ester (FAME) was identified based on retention time and compared with a standard mixture of 37 pure components.

#### 2.1.3. Elemental Analysis

Briefly, elemental analysis was conducted using an inductively coupled plasma optical emission spectrometry ICP-OES spectrometer (OPTIMA 7300 DV, Perkin Elmer, Waltham, MA, USA) a GemTip Cross-Flow II nebulizer (Perkin Elmer) equipped with an au-tosampler (SC-2 DX, Elemental Scientific Inc., Omaha, NE, USA). The detailed analytical procedure can be found in the study conducted by Lai et al. [28].

### 2.2. Chemometric Techniques

This work is the first of its kind to study the effect of the cheese production season on macro-components. The Tukey test was used to evaluate the statistical significance between the mean values of two production months for each macro-component. The selected threshold *p*-value for the test was *p* < 0.05. Fatty acid profiles and mineral content were analyzed from qualitative and quantitative perspectives. Concerning the classification task, in this study, an unsupervised method is compared with a supervised approach. PPCA is a statistical method that enables dimensionality reduction in high-dimensional datasets while accounting for noise and missing data [13]. More details on the technique are available in Supplementary Materials S1 (Section a). After performing PPCA, LDA was applied to the first two principal components to classify the data into three groups (one for each production season). LDA is used to find a linear combination of the principal components (PCs), thus constructing the boundaries where the Mahalanobis distances to the centroids of each class are equal [29]. Therefore, the linear boundaries maximize the separation between the groups. Once the sample scores were computed and LDA was performed, the presented model was employed to classify cheese samples. Partial Least Squares Discriminant Analysis (PLS-DA) was then employed in order to carry out a comparison between the two classifiers’ performances. After performing the classification, the estimation of % FAME from the mineral profile was performed using PPCA to assess its capability to manage missing data. PLS regression was also employed for comparative analysis in this investigation. It is important to specify that the PLS algorithm is unsuitable for handling missing data, meaning that the only variables completely characterized for each sample (i.e., those regarding mineral composition) were used as predictor variables for PLS-DA and PLS regression. A unity variance preprocessing step was conducted before executing the abovementioned algorithms.

### 2.3. Models’ Validation

In order to properly validate the PPCA and PLS-DA models in terms of classification, a Monte Carlo cross-validation [30] combined with external validation was employed, the process of which can be described through the following 4-step procedure:

- Step 1. Creation of the calibration set. The set is created by randomly selecting approximately 85% of the available complete samples. Further partial samples are added to the calibration set to constitute approximately 15 to 35% of the total calibration set.
- Step 2. Validation set construction. The remaining complete samples from step 1 are included for model cross-validation. Additional partial samples are included in the validation set, constituting approximately 30% to 65% of the total validation set. These samples are exclusively used for external validation, meaning that they are not replaced in the calibration set.
- Step 3. Calibrate the models. The entire calibration dataset is utilized to tune the PPCA and PLS-DA models.
- Step 4. Calculate the percentage of correctly classified samples (%CC). The PPCA and PLS-DA models are tested in reference to the validation samples, and their performance is assessed by determining their classification accuracy.

The presented methodology was applied to select the optimal number of Principal Components (PCs) and Latent Variables (LVs) for PPCA and PLS-DA, respectively. For each PC’s number (or LVs number), the abovementioned procedure was executed 20 times, and the models’ performance was assessed by computing the average %CC obtained for validation samples across all iterations. The optimal number of PCs and LVs was defined as the one for which the %CC was maximal. It is important to emphasize that in this procedure, the additional partial samples validated were not replaced across iterations and were never used for model calibration, thereby resembling as an external validation. Indeed, the models inferred the classes of these external partial samples in each iteration, while the calibration set comprised distinct sample groups each time it was randomly constructed. As a result, a different score estimation for external validation samples was obtained from each iteration as well, allowing for the quantification of parameter estimation sensitivity when a fraction of samples in the calibration set was replaced. Table 1 explains how the samples were distributed among the calibration and validation sets.

Concerning the assessment of PPCA and PLS in the quantitative estimation of % FAME, a Monte Carlo cross-validation method was employed to evaluate these models’ performances. Specifically, 20% of the complete samples were randomly selected for each production month and used for the internal validation step, while the remaining ones were retained for calibration [30]. Once calibrated, the PPCA and PLS models were tested via their application to the validation samples by predicting the % FAME using the samples’ mineral compositions. In both models, the reliability of the % FAME prediction was assessed using the coefficient of determination (*R*^2^) and the Root Mean Square of Errors in Cross-Validation (RMSECV). The entire procedure was carried out for a total of 20 iterations, and the overall *R*^2^ and RMSECV were calculated by averaging the values obtained in each iteration.

First, macrocomponent data were analyzed using the statistical software Minitab 21.1 (Minitab Inc., State College, PA, USA, 2022). Then, % FAME and mineral concentration (%*w*/*w*) analyses were carried out in the MATLAB^®^ environment. PPCA was carried out using the built-in functions provided by the Statistics toolbox, whereas validation was performed using an author-built routine. Conversely, LDA and PLS-DA were performed using the Classification Toolbox [31].

## 3. Results and Discussion

### 3.1. Pecorino Romano PDO Cheese Macro Composition Analysis

Table 2 shows the physicochemical composition and proteolysis indices (SN/TN, SN-TCA/TN, and SN-PTA/TN) for Pecorino Romano PDO cheese produced in January, April, and June. Based on the Tukey test, it was determined that the month of production statistically affected the fat and protein content of the cheese. In this study, it was observed that the fat content in cheese tends to decrease from winter to spring; then, it exhibits a significant increase in cheeses during the early summer. The protein content in cheese tends to exhibit a complementary behavior with respect to fat content, increasing in the cheese produced from January to April and then decreasing in the cheese produced in early summer.

As reported by other authors, this behavior is mainly caused by the natural variation in the fat/protein ratio in milk induced by the advancing production season [32]. The increase in the fat/protein ratio in sheep’s milk and cheese as the season progresses is a typical phenomenon occurring in sheep’s milk produced in Sardinia, and it is mainly linked to the stage of lactation, diet, and the rearing method practiced on the island. In particular, in the final stage of lactation, there is a progressive decrease in the volume of milk produced with a concentration of certain macro-components (particularly fat). Sheep’s milk, produced in the summer period, is characterized by a high fat content, which is generally not compensated for by an equal increase in protein content [7].

### 3.2. Multivariate Statistical Analysis on % FAME and Mineral Profiles

**Figure 1:**
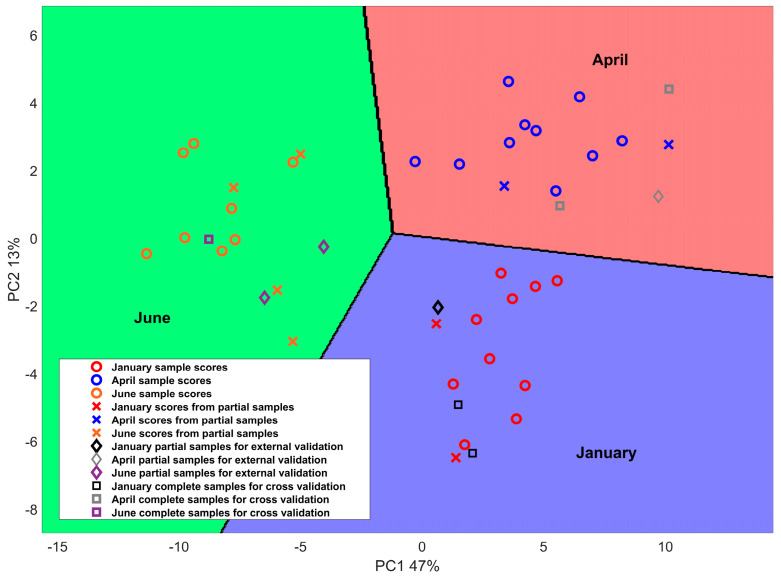
Score plot obtained from PPCA, and the relative boundaries obtained from LDA delimiting the regions to which the three different classes belong: Pecorino Romano produced in January, April, and June.

Many studies have investigated the influence of production season and reported its strong influence on the milk fatty acid profile and chemical composition of sheep milk products [33,34,35]. Indeed, it is well known that production season strongly affects the fatty acid composition of milk and, then of cheese, as a consequence of variations in animal feed, pasture availability, and the fatty acid composition of grass lipids [7,36]. Similarly, seasonal changes in animal diet have been found to affect the mineral composition profile of cheese [37]. Therefore, variations in % FAME and mineral concentration throughout the production season are expected to provide valuable information with which to develop a classifier that can be used to distinguish Pecorino Romano PDO cheese based on its manufacturing period. PPCA coupled with LDA was employed for such a task since PPCA is reliable in terms of reducing the complexity of a multivariate observed space and is efficient in reconstructing missing information. In contrast, LDA enables the discrimination of cheese samples based on the coordinates of the first two PPCA scores in a two-dimensional latent space. Using an example, Figure 1 reports the score plot of the first two principal components and the results of LDA classification for the calibration, cross-validation, and external validation samples obtained in an iteration. The first two PCs, which explained 47% and 13% of the total variance in the calibration set, were used to identify the main differences between samples.

**Figure 2:**
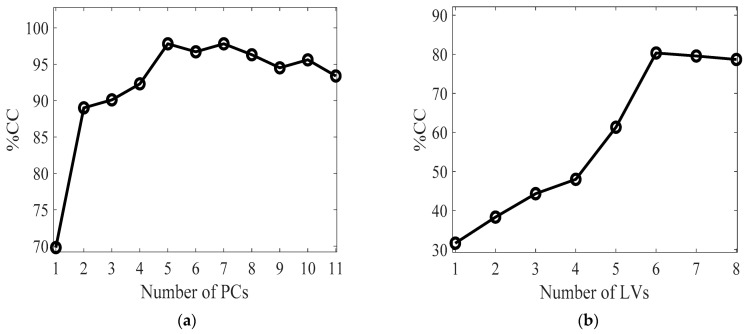
External cross-validation results. The performance regarding PPCA is shown for each PC (**a**), while the results of PLS-DA are presented with respect to the number of LVs (**b**). The accuracy of classification is expressed as the percentage of correctly classified samples.

It is evident that the application of the PPCA + LDA method to % FAME and mineral information yielded good classification results over different production times. Notably, cheeses produced during the winter and spring seasons show positive scores regarding PC1, while negative scores were reported for early summer. Concerning PC2, cheeses produced in April exhibited positive scores, while negative scores were observed for January. Regarding June, both positive and negative PC2 score values are reported in the plot. Similar results were obtained by Nudda et al., who reported a multivariate statistical analysis of the FA content in cheese samples produced in different seasons [8]. The same authors merged the early spring with the winter season and the late spring with the summer season, thus obtaining a binary analysis from the cheese seasonality point of view. The optimal number of PCs and LVs may vary depending on the overall dataset and the samples selected to calibrate the model. For this reason, cross-validation combined with external validation was employed to ensure the generalizability and reliability of the results. The results are reported in Figure 2.

Figure 2a shows the relationship between the number of principal components and the percentage of correct classifications (%CC) obtained in validation. It is evident that the maximum %CC, equal to 98%, was obtained from PPCA + LDA for five principal components, explaining 77% of the total variance. The model demonstrates satisfactory accuracy and robustness when a moderate number of PCs are used. Regarding the PLS-DA, the highest accuracy was achieved when using six LVs, resulting in a %CC of 80%. Accordingly, it appears that introducing partial information regarding % FAME into a PPCA model significantly improves the performance of the DA classifier. Conversely, using only mineral information coupled with a PLS-DA model leads to lower, yet still acceptable, performance accuracy in classification (Figure 2b).

### 3.3. PPCA Loading Analysis

**Figure 3:**
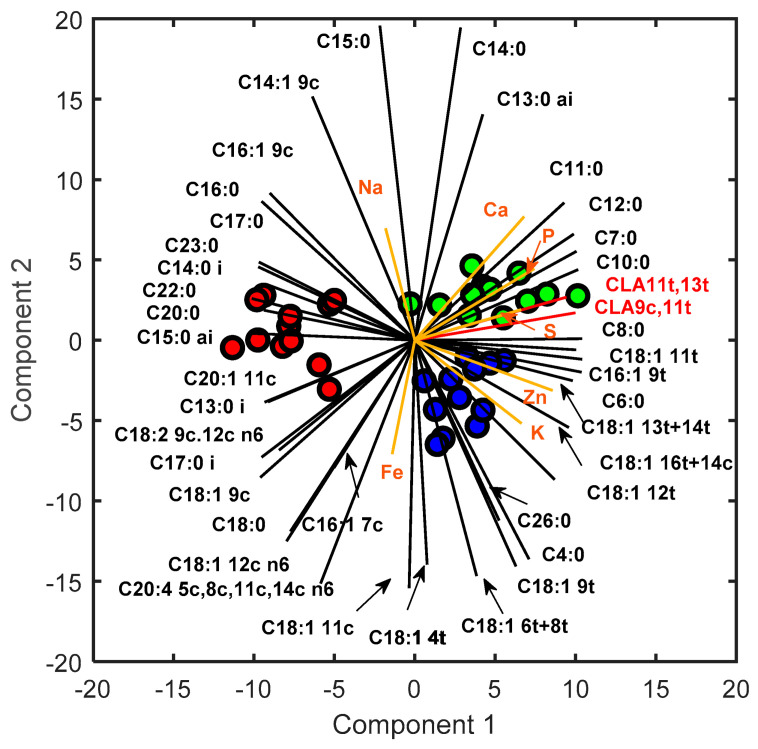
PPCA Biplot: Scores related to Pecorino Romano samples produced in January, April, and June are reported in blue, green, and red, respectively. Loadings related to the metabolites are reported in black, whereas those related to minerals are in orange.

In order to assess the impact of cheese production seasons on the content of individual fatty acids (FAs) and minerals, an analysis of loadings derived from PPCA and the identification of the most important variables with respect to constructing the first two PCs are reported in this section. The results of this analysis can provide valuable insights into which variables have the greatest impact on the observed variability in data. Particular attention has been paid to specific fatty acids, namely, rumenic acid, vaccenic acid, and omega-3 compounds, owing to their beneficial effects on human health. Figure 3 shows the biplot obtained using the PPCA for the calibration dataset.

A detailed description of the first and second component loading values is reported in Table S1 in Supplementary Material. Regarding the % FAME analysis, both PC1 and PC2 displayed positive loadings for rumenic acid, vaccenic acid, and omega3 compounds, such as alpha-linolenic acid (*C18:3 9c*,*12c*,*15c n3*) and eicosapentaenoic acid (*C20:5 5c*,*8c*,*11c*,*14c*,*17c n3*), indicating a positive correlation with April. Furthermore, high negative PC1 loadings were observed with regard to oleic acid, resulting in the determination of its positive correlation with June. Notably, these results are in line with those found in previous studies [7], even with respect to examining different food matrices like cow milk [38]. These findings can be explained via the combined effect of pasture quality and lactation stage [9]. In particular, the abundance of CLA and omega-3 compounds in cheese produced in the spring season is strongly associated with the wide availability, in the grazed pasture, of their precursor (*C18:3 9c*,*12c*,*15c n3*) during the grazing season [7]. In contrast, the increase in oleic acid during the late lactation stages may be due to the energy requirement induced by the worsening of pasture quality [8]. Particular attention has been paid to saturated fatty acids, which play a crucial role in defining the nutritional characteristics of food. Short-chain saturated FAs are strongly correlated with January; medium-chain saturated FAs are positively correlated with April. On the other hand, June is characterized by an abundance of long-chain saturated FAs. These loading trends highlight a clear dependence of the length of the saturated FA chain on the production season, which is probably due to the abovementioned reasons. Regarding the mineral loading analysis, S, P, and Ca showed positive loadings for both PC1 and PC2, indicating a positive correlation between these minerals and April as the month of cheese production, likely owing to the abundance of minerals during spring when the pasture quality is high. The higher presence of protein (caseins) in the cheese produced in April (Table 2) compared to the other periods could explain the high presence in the spring cheese of Ca and P, as they are key elements in the formation of the cheese protein matrix. K and Zn display positive PC1 and negative PC2 loadings, suggesting a positive correlation with January. For Fe, a weak dependence on the winter season was observed, while Mg did not strongly contribute to constructing the first two principal components as its loading values were low. Interestingly, Na loadings were negative in the first PC and positive in the second one, indicating that Na content is prevalent in June. A simple explanation for this result is that cheeses produced in June show a tendentially, though not significantly, higher salt content than the other cheeses (Table 2), as previously reported by other authors [7]. Regarding the mineral profile behavior, similar results were obtained in other studies [33,39,40].

### 3.4. Missing Data Reconstruction

**Figure 4:**
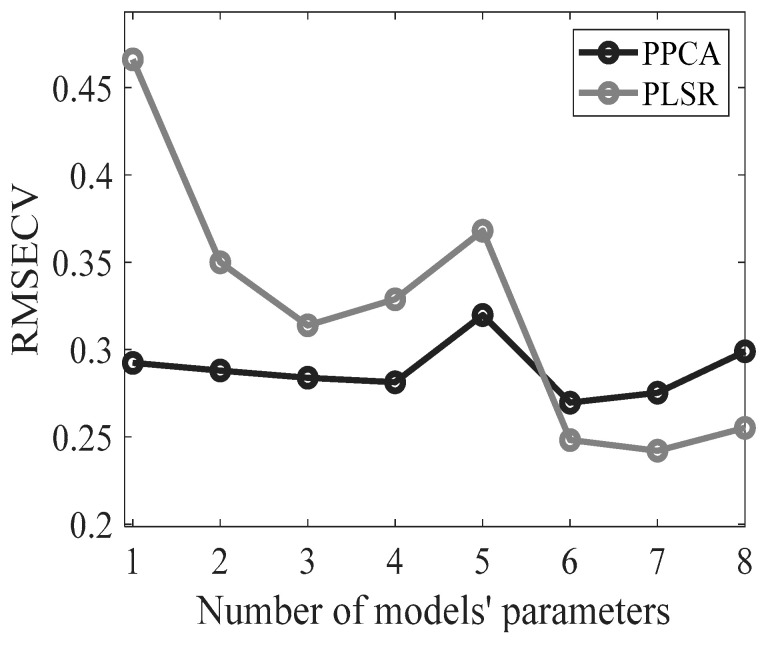
Root mean square error of cross-validation for PPCA and PLS models while varying the number of PCs and LVs, respectively.

The PPCA model’s effectiveness in reconstructing missing % FAME data was evaluated by comparing the estimated values with their corresponding experimental values from the cross-validation samples. During each iteration, % FAME information was removed from the validation samples, estimated by the model, and residuals were computed. Figure 4 compares the RMSECV for two models: PLS and PPCA. The *x*-axis shows the number of PCs/LVs involved in each model, while the *y*-axis shows the RMSECV values. According to the graph, the two models generally perform similarly, with PPCA performing better than PLS when a smaller number of components is considered. On the other hand, as the number of model parameters increases, the performance of PLS appears to improve, and PPCA continues to perform well. With six components, the performance of PLS regression is slightly superior to that of PPCA, resulting in a lower RMSE. This slight difference is maintained at higher dimensions.

Cross-validation analysis showed that the best data fit for the PPCA model was obtained with six PCs, resulting in an *R*^2^ value of 99.43%. On the other hand, the optimal PLS model was set with seven LVs, for which an *R*^2^ value of 99.53% was obtained. Thus, the performance of the models is comparable. It is worth noting that the presented PPCA model, like any model suitable for handling missing data, can be used as a classification and inferential tool [41]. Indeed, the same model can simultaneously provide informative score values that enable the classification of cheese samples and the reconstruction of the whole data matrix, thereby facilitating the prediction of missing values. Specifically, when the optimal number of PCs obtained in classification (i.e., 5 PCs) is employed to reconstruct data using PPCA, the result is an *R*^2^ value of 99.20% and an RMSE of 0.32 (Figure 3), meaning that good performance is maintained regarding missing data inference. In contrast, the PLS approach requires two different models, namely, PLS-DA and PLSR, to accomplish classification and inference tasks, respectively. In general, PPCA preserves high prediction performance, even when implemented with a parsimonious number of parameters, whereas PLSR achieves superior results only when model complexity is relatively high due to the involvement of several latent variables. Furthermore, when implementing a PPCA model, the computational cost may drastically increase as the number of PCs, even when the dataset is relatively small. Hence, the ability of the PPCA model to retain good performance with a limited number of PCs may facilitate the application of a probabilistic model, such as the one proposed in this work.

## 4. Conclusions

The application of a probabilistic model such as PPCA facilitates the monitoring of food quality when there are missing values in experimental observations. As reported herein, a comparative study was employed in order to assess the benefits offered by partial knowledge of the investigated variables when using a probabilistic model compared to a well-established multivariate model such as PLS. The case study reported in this work concerns the examination of the effect of seasonality on the fatty acid and mineral profiles for Pecorino Romano cheese, aiming to identify the key variables capable of discriminating cheese samples based on their production month. For classification purposes, such models were coupled with discriminant analysis algorithms, and Montecarlo cross-validation combined with external validation was employed to assess the classifiers’ reliability. The results showed that both models could effectively perform classification tasks, although PPCA showed particularly remarkable performance. Then, the ability of PPCA to handle missing data was exploited to reconstruct missing % FAME information, and PPCA was then compared with PLS regression, for which % FAME values were fitted by using only mineral compositions as predictor variables. The results showed that both models offer high predictive performance in the estimation of % FAME. Overall, it can be concluded that PPCA may be a valuable modelling strategy, as it simultaneously provides a classification tool and is capable of computing unknown information related to the presence of missing values, the latter of which is particularly common in the food industry, thus yielding robust methodology for analyzing food-omics data.

## Figures and Tables

**Table 1:** Number of samples in the calibration and validation sets for each month of cheese production.

	January		April		June	
	Number of Complete Samples	Number of Partial Samples	Number of Complete Samples	Number of Partial Samples	Number of Complete Samples	Number of Partial Samples
Calibration set	10	2	10	2	8	4
Validation set	2 ^a^	1 ^b^	2 ^a^	1 ^b^	1 ^a^	2 ^b^
Total	12	3	12	3	9	6

^a^ Samples employed to construct the internal cross-validation set. ^b^ Samples employed to construct external validation set.

**Table 2:** Macro composition and proteolysis indices for cheeses produced in three different seasons (presented as mean ± standard deviation).

Parameter	January	April	June
pH	5.07 ± 0.12 ^a,b^	5.10 ± 0.12 ^a^	5.01 ± 0.14 ^b^
Moisture (*w*/*w* %)	31.86 ± 1.43 ^a^	31.51 ± 0.97 ^a^	31.57 ± 1.05 ^a^
Fat/Dry matter (*w*/*w* %)	49.54 ± 1.31 ^b^	47.60 ± 1.40 ^c^	50.58 ± 1.28 ^a^
Protein/Dry matter (*w*/*w* %)	36.65 ± 0.94 ^b^	38.22 ± 1.12 ^a^	35.43 ± 1.10 ^c^
Fat/Protein ratio (-)	1.35 ± 0.05 ^b^	1.25 ± 0.05 ^c^	1.43 ± 0.06 ^a^
NaCl (*w*/*w* %)	4.48 ± 0.88 ^c^	4.59 ± 0.83 ^b^	5.02 ± 0.98 ^a^
Ash (*w*/*w* %)	7.20 ± 0.83 ^a^	7.48 ± 0.81 ^a^	7.57 ± 0.95 ^a^
SN/TN (%)	14.95 ± 2.25 ^a^	14.28 ± 3.11 ^a^	13.99 ± 2.07 ^a^
SN-TCA/TN (%)	21.21 ± 2.13 ^a^	11.92 ± 2.36 ^a^	11.22 ± 2.24 ^a^
SN-PTA/TN (%)	9.71 ± 2.51 ^a^	9.27 ± 1.79 ^a^	9.08 ± 1.89 ^a^

^a–c^ Statistically different means in the same row are annotated with different superscripts. DM: dry matter; SN: soluble nitrogen; TCA: trichloroacetic acid; PTA: phosphotungstic acid; TN: total nitrogen.

## Data Availability

PPCA validation software is available upon request from the first author. The data are not publicly available due to privacy.

## Supplementary Materials

The following supporting information can be downloaded at: https://www.mdpi.com/article/10.3390/metabo13070877/s1, S1. Probabilistic principal component analysis: Data projection onto latent space; Table S1: Loading analysis for probabilistic principal component analysis.
