# Supplementary material for: Investigation of Seasonal Variation in Fatty Acid and Mineral Concentrations of Pecorino Romano PDO Cheese: Imputation of Missing Values for Enhanced Classification and Metabolic Profile Reconstruction
Source: Metabolites. 2023 Jul 24;13(7):877. doi: 10.3390/metabo13070877 (PMC10386313; doi:10.3390/metabo13070877)
Supplement: Supplementary file 1 [file metabolites-13-00877-s001.zip › Supplementary materials.pdf]

Investigation of Seasonal Variation in Fatty Acid and Mineral Concentrations of Pecorino Romano PDO Cheese: Imputation of Missing Values for Enhanced Classification and Metabolic Profile Reconstruction

Leonardo Sibono [1], Massimiliano Grosso [1,*], Stefania Tronci [1], Massimiliano Errico [2], Margherita Addis [3], Monica Vacca [4], Cristina Manis [5] and Pierluigi Caboni [5]

[1] *Department of Mechanical, Chemical and Materials Engineering, University of Cagliari, Via Marengo 2, 09123 Cagliari, Italy*

[2] *Department of Green Technology, University of Southern Denmark, Campusvej 55, 5230 Odense, Denmark*

[3] *Agris Sardegna, Servizio Ricerca Prodotti di Origine Animale, Agris Sardegna, Loc., Bonassai, 07040 Sassari, Italy*

[4] *Servizio Ricerca Studi Ambientali, Difesa delle Colture e Qualità delle Produzioni, Viale Trieste, 09123 Cagliari, Italy*

[5] *Dipartimento di Scienze della vita e Ambiente. Cittadella Universitaria di Monserrato Blocco A, 09012 Monserrato, Italy*

* massimiliano.grosso@unica.it

S1. Probabilistic Principal Component Analysis: Data projection onto latent space

Probabilistic Principal Component Analysis seeks to relate a p-dimensional single observation vector $\underline{x}$ to a q-dimensional LVs vector $\underline{t}$ where q<p, thus mapping the original variable into a new space which offers a more parsimonious explanation of the dependences between observations. In such a model, LVs are assumed to be i.i.d and to follow a normal distribution. The p-dimensional error model $\underline{\varepsilon}$ is also considered, and it is assumed to behave like a gaussian noise $\underline{\varepsilon} \sim N(\underline{0}, \sigma^2 \underline{\underline{I}})$. Error is isotropic since the variances are assumed equal for all the p variables. Hence the PPCA model links the observation vector to a linear combination of the LVs, where noise term is taken into account as well (Tipping & Bishop, 1999):

$$\underline{x} = \underline{\underline{W}}\,\underline{t} + \underline{\mu} + \underline{\varepsilon} \qquad \text{(S.1)}$$

In Eq. A.1, $\underline{\underline{W}}$ is the loading matrix and $\underline{\mu}$ is the mean value for each observed variable. A linear combination of gaussian variables gives rise to a gaussian distribution for the observation vector $\underline{x} \sim N(\underline{\mu}, \underline{\underline{W}}\,\underline{\underline{W}}^T + \sigma^2 \underline{\underline{I}})$. Particularly, the use of an isotropic gaussian noise along with the factor model reported in Eq. 1 implies that the conditional probability distribution of the observation vector $\underline{x}$ given $\underline{t}$ is equal to:

$$\underline{x}|\underline{t} \sim N(\underline{\underline{W}}\,\underline{t} + \underline{\mu}, \sigma^2 \underline{\underline{I}}). \qquad (S.2)$$

Where the marginal distribution of $\underline{t}$ is conventionally defined by $\underline{t} \sim N(\underline{0}, \underline{\underline{I}})$. Nonetheless, when it becomes necessary to obtain model scores from experimental data (for instance, for classification purposes), it is crucial to calculate the estimated value of LVs from the observed data. This task can be achieved by applying Bayes' Theorem to $\underline{x}|\underline{t}$, $\underline{x}$ and $\underline{t}$ distributions, thus obtaining (Tipping & Bishop, 1999):

$$\underline{t}|\underline{x} \sim N(\underline{\underline{M}}^{-1} \underline{\underline{W}}^T (\underline{x} - \underline{\mu}), \sigma^2 \underline{\underline{M}}^{-1}). \qquad (S.3)$$

Where $\underline{\underline{M}}$ is a qxq matrix evaluated as follows:

$$\underline{\underline{M}} = \underline{\underline{W}}^T \underline{\underline{W}} + \sigma^2 \underline{\underline{I}}. \qquad (S.4)$$

The inference of the optimal loading matrix is performed by means of the EM algorithm, which maximises the log-likelihood function with respect to model parameters (i.e., σ^2 and $\underline{\underline{W}}$). In the E-step, the expected values of the LVs are estimated given the observed data and the current estimates of the model parameters. In the M-step, the model parameters are re-estimated by maximising the log likelihood function using the expected values of the LVs derived in the previous E-step. These two steps are repeated iteratively until the algorithm converges (Tipping & Bishop, 1999). Several convergence criteria are available in the literature (Nyamundanda et al., 2010). In this work, the convergence criterion requires that the EM algorithm be executed until the difference between two log-likelihood functions computed in two successive iterations is less than a tolerance value set to 0.001 or until the maximum number of iterations, set equal to 1000, is exceeded. It is important to specify that the maximum likelihood estimate of the loading matrix corresponds to the loading matrix given by conventional PCA, with the addition that the assessment of model uncertainty is available in such a probabilistic formulation.

Once the optimal loading values of the PPCA model have been identified, the next step is to project the data containing missing values onto the space of latent variables and then reconstruct the partial samples, given the scores obtained from the probability model. Firstly, observation vector $\underline{x}$ and the loading matrix for which the likelihood function is maximised $\underline{\underline{W}}_{ML}$ are split in two contributions:

$$\underline{x} = \begin{pmatrix} \underline{x}_k \\ \underline{N} \end{pmatrix} \qquad (S.5)$$

$$\underline{\underline{W}}_{ML} = \begin{pmatrix} \underline{\underline{W}}_{k,ML} \\ \underline{\underline{W}}_{m,ML} \end{pmatrix} \qquad (S.6)$$

Here, $\underline{N}$ corresponds to a rx1 vector correspondent to missing elements of the observation vector, which are identified as non-numerical values, whereas $\underline{\underline{W}}_{k,ML}$ is a (p-r)xq matrix which denotes the loading matrix partition related to the known variables (i.e. elemental composition) of the observation vector to project.

The quantiy r is the number of variables containing at least one missing value for a given sample. $\underline{\underline{W}}_{m,ML}$ denotes the loading matrix block associated with the missing information (i.e. %FAME). Therefore, a new $\underline{\underline{M}}_k$ matrix computed from $\underline{\underline{W}}_k$:

$$\underline{\underline{M}}_k = \underline{\underline{W}}_{k,ML}^T \underline{\underline{W}}_{k,ML} + \sigma^2 \underline{\underline{I}} \qquad \text{(S.7)}$$

$\underline{\underline{M}}$ matrix dimensionality is preserved when $\underline{\underline{W}}_k$ is used for $\underline{\underline{M}}_k$ calculation. The following step foresees the computation of the scores vecor $\underline{t}_k$ made up of the contribution of the sole loading related to known variables for each specific sample:

$$\underline{t}_k = \underline{\underline{M}}_k^{-1} \underline{\underline{W}}_{k,ML}^T \left(\underline{x}_k - \underline{\mu}_k \right) \qquad \text{(S.8)}$$

Where $\underline{\mu}_k$is a (p-r)x1 vector of variables average values. The expression reported in eq. A.7 is not the final form of score vector, as it only considers the contribute of non-missing variables on the projection onto the latent space. Therefore, $\underline{t}_k$ is employed to obtain the reconstructed data (Tipping & Bishop, 1999):

$$\underline{\hat{x}} = \underline{\underline{W}}_{ML} \left(\underline{\underline{W}}_{ML}^T \underline{\underline{W}}_{ML} \right)^{-1} \underline{\underline{M}}\, \underline{t}_k + \underline{\mu} \qquad \text{(S.9)}$$

It is important to specify that $\underline{\underline{W}}_{ML}$is not orthogonal and thus not optimal (Tipping & Bishop, 1999). $\underline{\underline{W}}_{ML}$ is then expressed in therms of its singular value decomposition:

$$\underline{\underline{W}}_{ML} = \underline{\underline{U}}\, \underline{\underline{L}}\, \underline{\underline{V}}^T \qquad \text{(S.10)}$$

Where $\underline{\underline{U}}$ is a pxq matrix of orthonormal column vectors, $\underline{\underline{L}}$ is the qxq diagonal matrix of singular values, and $\underline{\underline{V}}$ is a qxq orthogonal matrix. $\underline{\underline{U}}$ matrix represents the orthogonalised form of loading matrix obtained from maximum likelihood estimation, and corresponds to the loadings obtained by conventional PCA. Hence the final expression of score matrix obtained from the overall contribution of both known and reconstructed information is given as follows:

$$\underline{t} = \underline{\underline{U}}^T \left(\underline{\hat{x}} - \underline{\mu} \right) \qquad \text{(S.11)}$$

The first two elements of $\underline{t}$ score vector were plotted in figure 1, for classification purposes.

Table S1. Loading values on first and second PCs obtained by PPCA

Variable	Loading on PC1	Loading on PC2	Variable	Loading on PC1	Loading on PC2	Variable	Loading on PC1	Loading on PC2
'C4:0'	0.109305	-0.20962	'C18:1 5t'	0.0267	-0.02454	'CLA 9t,11c'	-0.11032	0.0479
'C6:0'	0.159599	-0.03058	'C18:1 6t + C18:1 8t'	0.05921	-0.22573	'CLA 10t,12c+C21:0'	-0.05506	-0.01006
'C7:0'	0.154653	0.085226	'C18:1 9t'	0.09675	-0.21659	'CLA 9c,11c'	0.14933	0.01322
'C8:0'	0.160066	0.001488	'C18:1 10t'	0.08931	0.03098	'CLA 12t,14t + CLA 11c 13c'	0.10765	-0.06803
'C10:0'	0.156294	0.067634	'C18:1 11t'	0.1601	-0.01826	'CLA 11t,13t'	0.145	0.04116
'C11:0'	0.142946	0.131658	'C18:1 12t'	0.13384	-0.13339	'CLA 9t,11t'	0.04365	0.11291
'C12:0'	0.152066	0.101636	'C18:1 13t + C18:1 14t'	0.15111	-0.03878	'C20:2 11c,14c n6'	0.0392	-0.00811
'C13:0 i'	-0.143654	-0.05934	'C18:1 9c'	-0.14782	-0.13137	'C20:3 5c,8c,11c'	0	0
'C13:0 ai'	0.065134	0.216256	'C18:1 15t + C18:1 10c'	-0.07415	0.01776	'C22:0'	-0.15589	0.03995
'C14:0 i'	-0.141302	0.053299	'C18:1 11c'	-0.00544	-0.23734	'C20:3 8c,11c,14c n6'	0	0
'C14:0'	0.04377	0.299376	'C18:1 12c n6'	-0.1227	-0.19239	'C20:3 11c,14c,17c n3'	0	0
'C15:0 i'	-0.001255	0.01442	'C18:1 13c'	0.14177	-0.02881	'C20:4 5c,8c,11c,14c n6'	-0.09007	-0.23291
'C15:0 ai'	-0.157415	0.006647	'C18:1 16t + C18:1 14c'	0.14731	-0.08363	'C23:0'	-0.14974	0.07024
'C14:1 9c'	-0.097979	0.233402	'C18:2 9t,12t n6'	0.05362	0.11814	'C24:0'	-0.13884	0.02298
'C15:0'	-0.03347	0.301288	'C18:2 9c,13t'	0.14355	0.00854	'C20:5 5c,8c,11c,14c,17c n3'	0.07998	0.048
'C16:0 i'	-0.069347	-0.04938	'C18:2 9c,12t + C18:2 8t 12c n6'	0.1388	-0.04486	'C24:1 15c'	-0.09548	-0.00974
'C16:0'	-0.146491	0.133046	'C18:1 16c'	0.12804	-0.00779	'C26:0'	0.08071	-0.17262
'C17:0 i '	-0.129969	-0.10527	'C18:2 9t,12c n6'	-0.05811	0.08004	'C22:5 7c,10c,13c,16c,19c'	-0.09787	0.02091
'C16:1 9t'	0.154378	-0.00921	'C18:2 11t,15c n3'	0.1491	0.00581	'C22:6 4c,7c, 10c,13c,16c,19c n3'	-0.09954	-0.05648
'C16:1 7c'	-0.077373	-0.12222	'C18:2 9c,12c n6'	-0.14649	-0.11195	'Ca'	0.11432	0.06848
'C17:0 ai'	0.01944	0.092416	'C18:2 9c,15c n3'	0.10441	-0.08581	'Mg'	0.04028	-0.00029

'C16:1 9c'	-0.138848	0.14086	'C20:0'	-0.14442	0.02877	'Na'	-0.02792	0.10717
'C17:0'	-0.149132	0.074825	'C18:3 6c,9c,12c n6'	-0.00718	0.05701	'K'	0.10207	-0.07966
'C18:0 i'	-0.071596	-0.02956	'C20:1 9c '	-0.08928	0.08684	'P'	0.10454	0.11838
'C17:1 10c'	0	0	'C20:1 11c'	-0.14044	-0.05902	'S'	0.10164	0.02933
'C18:0'	-0.118953	-0.183	'C18:3 9c,12c,15c n3'	0.1131	0.01354	'Zn'	0.13186	-0.04806
'C18:1 4t'	0.011864	-0.2148	'CLA 9c,11t'	0.15374	0.02622	'Fe'	-0.02162	-0.10903
